# Inverted papilloma originating from the left ethmoid sinus invading the nasal cavity bilaterally via the frontal sinus septum^☆^

**DOI:** 10.1016/j.bjorl.2015.03.017

**Published:** 2015-09-09

**Authors:** Zhao Wei Gu, Yun Xiu Wang, Zhi Wei Cao

**Affiliations:** Department of Otorhinolaryngology, Shengjing Hospital of China Medical University, Liaoning Province, China

## Introduction

Inverted papillomas (IPs) are common benign sinonasal tumors characterized by a high risk of recurrence and potential for malignancy. The lateral nasal wall and middle meatus are the most common sites for IP to arise, but they can develop from any part of the Schneiderian membrane in the nose and paranasal sinuses. IPs are usually confined to one side of the nasal cavity; bilateral involvement of the nose and paranasal sinuses is extremely rare, with only a few cases reported in the medical literature in English-language.

This report presents a case of a large IP that originated from the left ethmoid sinus and spread to both sides of the nasal cavity through the frontal sinus septum. This type of diffusion is extremely rare. The tumor was successfully treated with a combined endoscopic and external approach. The need for a better understanding of the pathogenesis of bilateral involvement is discussed, as well as the importance of surgical technique when treating this rare disease.

Prior informed consent was obtained from the patient. This study was approved by the ethics committee of Shengjing Hospital, affiliated with China Medical University.

## Case report

**Figure 1:**
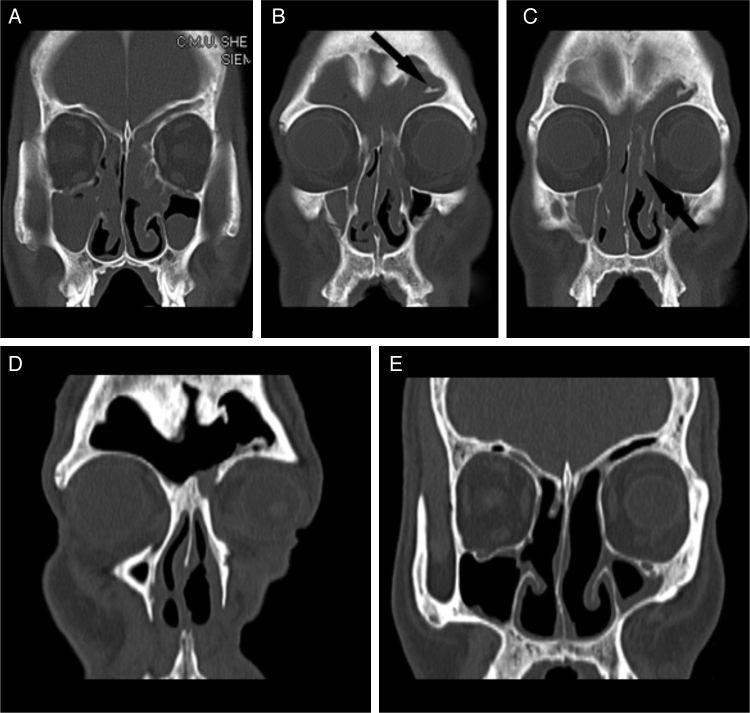
(A–C) Preoperative coronal computed tomography (CT) scan showing: (A) a hyperdense mass occupying both nasal cavities and the bilateral frontal, bilateral ethmoid, and bilateral maxillary sinuses; (B) the well-pneumatized frontal sinus, destruction of the frontal sinus septum, a bone septum on the lateral wall of the left frontal sinus (black arrow), and a slight deviation of the nasal septum to the right; and (C) the enlarged frontal ostium and focal hyperostosis in the left ethmoid sinus (black arrow). (D and E) Postoperative coronal CT scan after complete removal of the mass. There was no sign of recurrence two years after surgery.

A 53-year-old man presented to the hospital with a four-year history of progressive bilateral nasal obstruction and hyposmia with chronic headache. There was no history of epistaxis. He had undergone a nonendoscopic left endonasal polypectomy at another institution. The patient was otherwise healthy. Physical examination revealed bilateral intranasal polypoidal masses that bled readily on contact. The nasal septum had no perforation and deviated to the right side. Sinus computed tomography showed a hyperdense image occupying both nasal cavities and the bilateral frontal, bilateral ethmoid, bilateral maxillary, and left sphenoid sinuses (Fig. 1A and B). In addition to the well-pneumatized frontal sinus, there was destruction of the frontal sinus septum, a bone septum on the lateral wall of the left frontal sinus, slight deviation of the nasal septum to the right (Fig. 1B), an enlarged frontal ostium, and focal hyperostosis in the left ethmoid sinus suggesting the origin of the IP^1^, ^2^ (Fig. 1C). There were no signs of bone lysis. Bilateral outpatient biopsy was performed, and the histopathologic report of both specimens confirmed an IP. The patient underwent endoscopic sinus surgery under general anesthesia. The tumor appeared to have arisen from the left ethmoid sinus. It involved the frontal sinus and skull base, and extended around the left maxillary antrum. The tumor on the left side penetrated into the right nasal cavity through the septum of the frontal sinus and the right aperture of the sinus frontalis. A cyst was visible in the left maxillary sinus. Complete endoscopic mucosal stripping with resection of the middle and upper turbinates, maxillary sinus antrostomy, and ethmoidectomy were performed in the left nasal cavity, while ethmoidectomy with maxillary sinus antrostomy was performed on the right. The tumor and the bilateral frontal sinuses were resected via an endoscopic left Lynch incision. The permanent pathology report revealed a benign IP. Because the frontal sinuses were involved and there was no evidence of malignant transformation, the tumor was stage III according to the Krouse staging system.^3^ The postoperative recovery was uneventful, and no recurrence was observed at the two-year follow-up examination (Fig. 1D and E).

## Discussion

Bilateral involvement of the nose and paranasal sinuses is extremely rare in patients with IP, with only a few cases reported in the English-language literature. An IP originating from one side of the ethmoid sinus can lead to the development of bilateral IPs via the nasal septum or other multicenter diffusion pathways.^4^ To the best of the author's knowledge, the present report describes the first case of an IP originating from the left ethmoid sinus and involving both sides of the nasal cavity via the frontal sinus septum. This type of diffusion is common in primary frontal sinus IPs.^5^ In this case, the frontal sinus ostium was large, which may have facilitated the tumor's invasion of the frontal sinus. Additionally, the frontal sinus is well-pneumatized and the frontal sinus septum is often thin or missing, allowing growth of the tumor into the contralateral frontal sinus through the frontal sinus septum.

The preferred treatment of IPs is complete wide resection. Success is fully dependent upon complete resection of the IP and careful stripping of the underlying diseased mucosa. Successful endoscopic removal of IPs has been described by many authors, and its advantages include the avoidance of a facial incision or an external scar, a shorter hospital stay, less facial swelling, decreased postoperative pain, and reduced blood loss.^6^

An endoscopic modified Lothrop procedure (EMLP), which involves complete removal of the bilateral frontal sinus floor and superior septum, provides sufficient endoscopic access for most frontal sinus IPs and may obviate the need for an open approach.^1^, ^5^, ^7^ EMLP has been used successfully in the treatment of IPs involving the frontal sinus with attachment to the medial wall, posterior wall, or intersinus septum,^7^ and is highly recommended for the endoscopic treatment of bilateral and/or multifocal frontal sinus lesions.^1^

However, EMLP has several disadvantages in patients with disease extending to the lateral wall of a well-pneumatized frontal sinus or the anterior wall, as well as in cases of multifocal attachment.^5^, ^8^ In the present patient, the bilateral frontal sinuses were well-pneumatized, the lesions involved the lateral wall of the frontal sinus, and a bone septum was present on the lateral wall of the left frontal sinus. Therefore, EMLP was deemed therapeutically inappropriate. Instead, a combined endoscopic and external approach was chosen to ensure that the tumor was completely resected. The postoperative recovery was uneventful.

## Final remarks

This report represents the first known case of an IP originating from the left ethmoid sinus and involving both sides of the nasal cavity via the frontal sinus septum. It is speculated that in a well-pneumatized frontal sinus with a larger frontal ostium, a unilateral ethmoid IP may spread to the contralateral nasal cavity. This study supports the conclusion that surgical resection is the preferred treatment for IP.

## Conflicts of interest

The authors declare no conflicts of interest.
